# Deep learning detection and quantification of pneumothorax in heterogeneous routine chest computed tomography

**DOI:** 10.1186/s41747-020-00152-7

**Published:** 2020-04-17

**Authors:** Sebastian Röhrich, Thomas Schlegl, Constanze Bardach, Helmut Prosch, Georg Langs

**Affiliations:** 1 Department of Biomedical Imaging and Image-guided Therapy, Medical University of Vienna, Vienna, Austria; 2 Contextflow GmbH, Vienna, Austria; 3 Computational Imaging Research Lab, Department of Biomedical Imaging and Image-guided Therapy, Medical University of Vienna, Vienna, Austria

**Keywords:** Deep learning, Pneumothorax, Thorax, Tomography (x-ray computed), Triage

## Abstract

**Background:**

Automatically detecting and quantifying pneumothorax on chest computed tomography (CT) may impact clinical decision-making. Machine learning methods published so far struggle with the heterogeneity of technical parameters and the presence of additional pathologies, highlighting the importance of stable algorithms.

**Methods:**

A deep residual UNet was developed and evaluated for automated, volume-level pneumothorax grading (*i.e.,* labelling a volume whether a pneumothorax was present or not), and pixel-level classification (*i.e.,* segmentation and quantification of pneumothorax), on a retrospective series of routine chest CT data. Ground truth annotations were provided by radiologists. The fully automated pixel-level pneumothorax segmentation method was trained using 43 chest CT scans and evaluated on 9 chest CT scans with pixel-level annotation basis and 567 chest CT scans on a volume-level basis.

**Results:**

This method achieved a receiver operating characteristic area under the curve (AUC) of 0.98, an average precision of 0.97, and a Dice similarity coefficient (DSC) of 0.94. This segmentation performance resulted to be similar to the inter-rater segmentation accuracy of two radiologists, who achieved a DSC of 0.92. The comparison of manual and automated pneumothorax quantification yielded a Pearson correlation coefficient of 0.996. The volume-level pneumothorax grading accuracy was evaluated on 567 chest CT scans and yielded an AUC of 0.98 and an average precision of 0.95.

**Conclusions:**

We proposed a deep learning method for the detection and quantification of pneumothorax in heterogeneous routine clinical data that may facilitate the automated triage of urgent examinations and enable treatment decision support.

## Key points

- Pneumothorax is an important pathology to be included in applications that are designed to triage urgent imaging examinations.
- Heterogeneity in routine clinical data may be overcome by utilising deep learning methods.
- Additional automated quantification of pneumothorax volume correlates well with manual volumetric assessment, but is less time-consuming.

## Background

Automated triage of patients in radiology is a rapidly developing machine learning application with the goal of early detection of urgent pathologies [1, 2]. One such pathology is a pneumothorax, the relevance of which is reflected by its frequency and the possibility of severe complications. Regardless of its aetiology, a tension pneumothorax may develop and lead to a rapid deterioration of the patient. Furthermore, for spontaneous, traumatic, and iatrogenic pneumothoraxes, there are different and specific treatment suggestions, all of which depend on a multitude of factors [3]. Whereas some pneumothoraxes may be treated conventionally (*i.e.,* by observation), others will need to be aspirated with a needle or require the placing of a chest tube to relieve the pressure, the latter being frequently conducted in trauma patients [3]. Therefore, for radiological triaging systems that aim to provide a thorough evaluation of a patient's condition, it will be necessary to include pneumothorax and report on its therapy-relevant features.

Next to clinical symptoms, the treatment decision can be partially supported by the pneumothorax extent or volume as measured on chest radiographs or computed tomography (CT) scans [4–6]. When the cause is spontaneous, a ‘large’ pneumothorax is defined as larger than 3 cm or than 15% of the volume of the hemithorax and requires aspiration [7]. Although in trauma patients, chest tubes are frequently placed, recent publications have shown increasing evidence that supports the conservative treatment of traumatic pneumothoraxes [8], with one study suggesting conservative treatment for pneumothoraxes after blunt trauma with a volume lower than 30 mL [5]. Thus, there is an ongoing debate about what the appropriate management should encompass as the relevant volume or threshold of free air that mandates the placing of a chest tube has not been exactly defined, as yet.

To estimate the size of a pneumothorax, different modalities can be used, such as chest radiography and ultrasound, with CT scans constituting the standard of reference [3]. While a rough estimate can be quickly done by measuring the distance between the pleural leaflets perpendicular to the lung surface and thoracic wall, the quantification of the volume requires more sophisticated approaches. The manual segmentation of a pneumothorax is not feasible in the clinical routine and is too time-consuming for a large number of cases, thus necessitating automation [9].

Some publications have shown the possibility of automatically quantifying the volume of pneumothorax, in both adults [9, 10] and paediatric patients [11]. These studies have used specific functions in a multistep approach to achieve the final volume estimate. However, such algorithms may be prone to bias through concurrent pathologies [12]; consequently, scans with these pathologies were excluded in one of these studies [9]. In these studies, pneumothorax quantification on chest CT was performed using different methods, obtaining a sensitivity of 100%; however, specificity ranged from 10 to 100%, with low values in the cases of small pneumothorax or concurrent pathologies such as emphysema and bullae [10, 13].

The recent advances of machine learning applications in radiology have resulted in several publications about the automatic detection of pneumothorax in large-scale, clinical, and routine chest x-ray datasets. By applying deep-learning methods, it was possible to overcome the heterogeneity of technical parameters and the variability due to concurrent pathologies that would otherwise hamper accurate detection [14, 15].

For CT scans, previous studies have relied on the combination of specific functions and machine-learning steps to segment the lungs in the presence of pneumothorax and other pulmonary pathologies [12]. However, there are no publications that have used deep-learning exclusively for pneumothorax detection and quantification on chest CT scans. Therefore, the goal of this study was to develop and evaluate the performance of a deep learning algorithm to triage emergency and routine chest CT scans with heterogeneous pathologies and acquisition parameters based on pneumothorax presence and size. This may enable urgent cases to be put at the top of the worklist and to aid treatment decisions.

## Methods

**Fig. 1:**
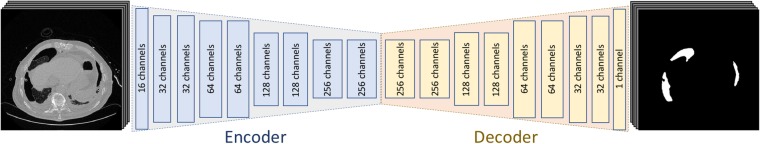
Neural network architecture. The proposed automated pneumothorax classification model is based on a UNet architecture that comprises an encoder-decoder architecture

The local ethics committee of the Medical University of Vienna approved the retrospective analysis of the imaging data for the study (approval number 1154/2014). Here, we present a pneumothorax classifier that can detect a pneumothorax at the pixel level. In addition, we use the same classifier for the volume-level pneumothorax-grading task. More specifically, for volume-level pneumothorax grading, we did not train a separate model but used the pixel-level classification results by simply aggregating the pixel-level predictions to derive a volume-level score. In Fig. 1, we show an illustration of the proposed automated method.

### Imaging data

We collected all chest CT scans from the clinical routine over a timeframe of 2.5 years from 2013 to 2015 and generated labels for ‘pneumothorax’ and ‘no pneumothorax’ based on the radiological reports and visual verification by a radiologist with three years of subspecialty training in thoracic radiology. In total, chest CT scans of 610 unique patients were included, were included, from which 43 were randomly chosen for pixel-level segmentation and 567 were randomly chosen for volume-level grading. Patient age ranged from 1 to 92 years, with a mean age of 54 years (standard deviation 19 years), 384 males (63% ) and 226 females (37%). All images were acquired on one of the three following scanners: Somatom Cardiac Sensation 64 (Siemens Medical Solutions, Forchheim, Germany); Somatom Definition Flash (Siemens Medical Solutions, Forchheim, Germany); or Brilliance CT 64 (Philips Medical Systems, Cleveland, OH, USA).

There were no exclusion criteria, resulting in a wide variety of pathologies. The most common main pulmonary diagnoses were post-surgical complications (48/610, 7.9%), malignant neoplasm of unspecified part of the bronchus or lung (37/610, 6.0%), chronic obstructive pulmonary disease (31/610, 5.0%), secondary malignant neoplasm of the lung (29/610, 4.8%), pneumonia (25/610, 4%), acute respiratory failure (24/610, (3.9%) and lung transplantation (23/610, 3.8%), with other pulmonary diagnoses occurring in less than 1% of cases.

### Pixel-level classification

For training and evaluation of the *pixel-level classification* model, we randomly selected 43 chest CT scans with a pneumothorax from the clinical routine CT scans. Only axial slices in the inspiratory phase that were reconstructed using lung kernels were included. Technical specifications are provided in Table 1.

**Table 1:** Technical specifications for the pixel- and volume-level cases

	Pixel-level cases	Volume-level cases
Slice thickness (mm)	1, 1.5, 3	1, 1.5, 2, 3
Pixel spacing (mm)	0.51 × 0.51 to 0.84 × 0.84	0.35 × 0.35 to 0.97 × 0.97
Tube voltage (kV)	100, 120	80, 100, 120, 140
Exposure (mAs)	10 to 284	30 to 382
Filter type	WEDGE_2, WEDGE_3, YB, no filter	FLAT, WEDGE_2, WEDGE_3, YB, D, no filter
Convolution kernel	B60f, B70f, YB	B60f, B60s, B70f, I70f\2, I70f\3, YB, D

A radiologist with 5 years of experience in thoracic CT provided pixel-level manual annotations of pneumothorax regions for single, axial slices using ITK-SNAP [16]. Axial slices were selected to represent a good coverage of the relevant occurrences of pneumothorax. The manual generation of annotations is very time-consuming and not practical for every slice of a CT scan when working on more than a few scans. Our solution was to annotate one slice every fifth to tenth slice and to automatically interpolate intermediate slices. In addition, the first (cranial) and last (caudal) slice (*z*-position) of each pneumothorax per volume was labelled with a specific label and interpolations were restricted to those slice ranges. For the interpolation (*i.e.,* approximation of the missing annotations) of each interposed slice, the *x*/*y* in-plane positions of boundary pixels were linearly interpolated along the *z*-axis. All pixels that were located on the resulting boundary and within the boundary were automatically annotated with the pneumothorax label. These additional labels were used only for model training but were not used as ground-truth labels for model evaluation.

**Fig. 2:**
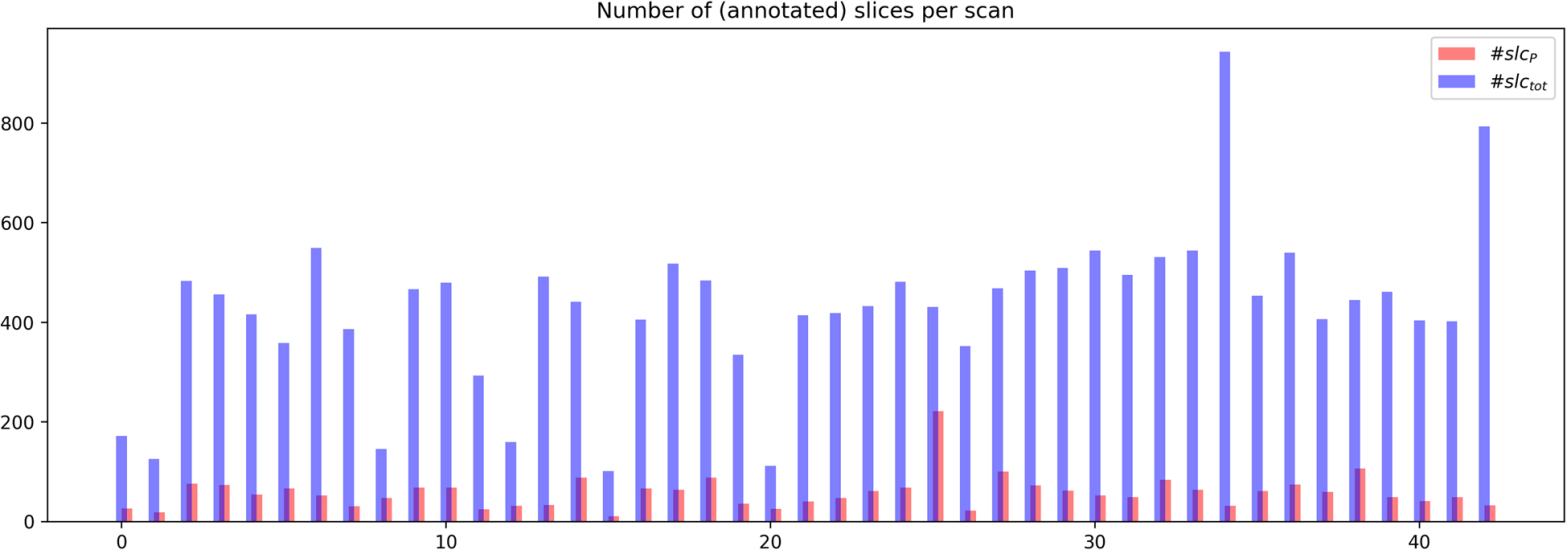
Distribution of the annotated data. Blue bars (#slc_tot_) show the total number of slices for each scan, and red bars (#slc_*p*_) show the annotated number of slices for each scan

In total, we had 2487 annotated axial slices with, on average, 57 annotated slices per scan, whereas, on average, 1.86% of all pixels of an image were assigned the ‘pneumothorax’ class label. We applied a statistical model that handles distinct *class imbalances*. Figure 2 provides an overview of the data statistics of the annotated data.

### Volume-level grading

Volume-level pneumothorax grading was performed using the pixel-level pneumothorax classifier. Therefore, for volume-level grading, we did not require additional training data. For only the final evaluation of the volume-level pneumothorax detection accuracy, we randomly selected 567 chest CT scans, in which the occurrence of pneumothorax was labelled at the *volume-level*. There were 167 (29%) volumes that contained and 400 (71%) volumes that did not contain a pneumothorax (see Table 1).

For both the pixel- and volume-level classifications, only unique patients were selected. For data pre-processing, we applied a lung window with a grey value range of [− 1000, 400] and mapped this grey value representation to the range [0, 1].

### Automated classification method

The algorithm classifies every pixel position within two-dimensional axial images extracted from chest CT volumes for the differentiation between pneumothorax and background regions. We applied semantic segmentation [17], a state-of-the-art, machine-learning method that utilises deep neural networks (known as deep learning), to compute pixel-level classification maps from clinical chest CT scans. To build this automated model, supervised learning on annotated training data, comprising clinical chest CT scans and corresponding pixel-level annotations of pneumothorax, was utilised.

The semantic segmentation technique is based on a neural network architecture that comprises two components: an encoder network and a decoder network. The encoder maps input images to a meaningful, low-dimensional, abstract representation, which can be interpreted as a compression of the original signal (image) that retains the most relevant and informative signal components. The subsequent decoder network maps this compact representation to a map of class labels so that each pixel of the input image has a corresponding class label prediction. Both the parameters of the decoder network, which yields an accurate mapping of the low-dimensional, abstract representation of class labels, and the parameters of the encoder network, which yields the most informative inputs for the decoder, are automatically learned by the learning algorithm during training, based solely on pairs of input images and corresponding target labels. Both neural network components are trained simultaneously. Specifically, we implemented a UNet-based image segmentation network [17] and used residual units [18] as feature extractors. An overview of the utilised UNet architecture is shown in Fig. 1.

The model was trained for 3500 epochs (*i.e.,* full passes through all training volumes) using a cross-entropy objective. The model was trained and evaluated on a TitanX graphics processing unit (Nvidia, Santa Clara, CA, USA) with Python 2.7 and Tensorflow [19] (version 1.3), and with the deep learning toolkit (DLTK [20]) for medical imaging.

### Experimental setup

For model training and evaluation, we split the data into a training set (27 volumes), a validation set (7 volumes), and a test set (9 volumes). Model training was performed on the training set. Hyper-parameter tuning and model selection was performed on the validation set. The test set was only used once, namely, for the final evaluation of the actual model accuracy.

### Execution time

For all volumes of the test set, we measured the execution time of processing all raw pixel values of a full CT scan to pixel-level classifications of the full volumes as belonging or not to pneumothorax. The execution time per CT scan is the time that our algorithm takes for transforming a full raw volume into a volume of pixel-level classifications. We report average, minimum, and maximum execution times over all volumes of the test set.

### Statistical analysis

We evaluated the performance of the model for pixel-level classification accuracy, volume-level grading accuracy, and interrater variability.

#### Pixel-level classification accuracy

We evaluated the pixel-level segmentation accuracy, *i.e.,* the accuracy of correctly classifying individual pixels as belonging or not belonging to pneumothorax, of the trained classifier on the test set, which comprises only chest CT scans that were not used during model training. We parsed each full volume of the test set and computed the Dice coefficient score (DSC), which is defined as

$$ DSC=\frac{2\cdot precision\cdot recall}{precision+ recall}=\frac{2\cdot {t}^{+}}{2\cdot {t}^{+}+{f}^{+}+{f}^{-}} $$

where *t*^+^is the number of true positive pixels, *f*^+^is the number of false-positive pixels, and *f*^−^ is the number of false-negative pixels. In addition, we computed precision, recall, and specificity values. Furthermore, we plotted the receiver operating characteristic (ROC) and precision-recall curves and provide the corresponding area under the curve (AUC) and average precision values.

The quantification of the total area of a pneumothorax within an axial slice of a chest CT scan was computed based on the corresponding pixel-level pneumothorax segmentations. We evaluated the pneumothorax quantification accuracy by aggregating the segmented pneumothorax pixels. Pneumothorax areas are approximated as the number of pixels classified (or annotated) as a pneumothorax per axial slice. We plotted correlation plots and calculated Pearson correlation coefficient, *R*^2^, and the corresponding two-tailed *p* value.

#### Volume-level grading accuracy

We evaluated the volume-level pneumothorax detection accuracy, *i.e.,* the accuracy of correctly classifying a whole chest CT scan as having or not having a pneumothorax, on chest CT scans with volume-level pneumothorax grading. We computed DSC, precision, recall, and specificity values. Furthermore, we plotted ROC and precision-recall curves and provide corresponding AUC and average precision values. Pixel-level classification results were used for volume-level pneumothorax grading. More specifically, pixel-level pneumothorax class probabilities were used to perform volume-level pneumothorax grading. To evaluate the volume-level pneumothorax grading accuracy, we parsed each full volume and assigned those pixels the pneumothorax label that exceeded the threshold (in terms of output probability), which corresponded to the value at the Youden Index or optimal cut-off point of the corresponding AUC or precision-recall curve.

#### Interrater variability

In total, 86 axial slices from two chest CT scans were independently annotated by two radiologists with 4 and 5 years of experience with thoracic CT. Based on this data, the interrater variability between two independent annotators was evaluated to approximate the maximal achievable segmentation accuracy. The consensus of both radiologists with regard to pneumothorax identification on the pixel level was evaluated based on the DSC and based on the correlation of the slice-wise pneumothorax quantification.

## Results

### Pixel-level classification

#### Segmentation accuracy

**Fig. 3:**
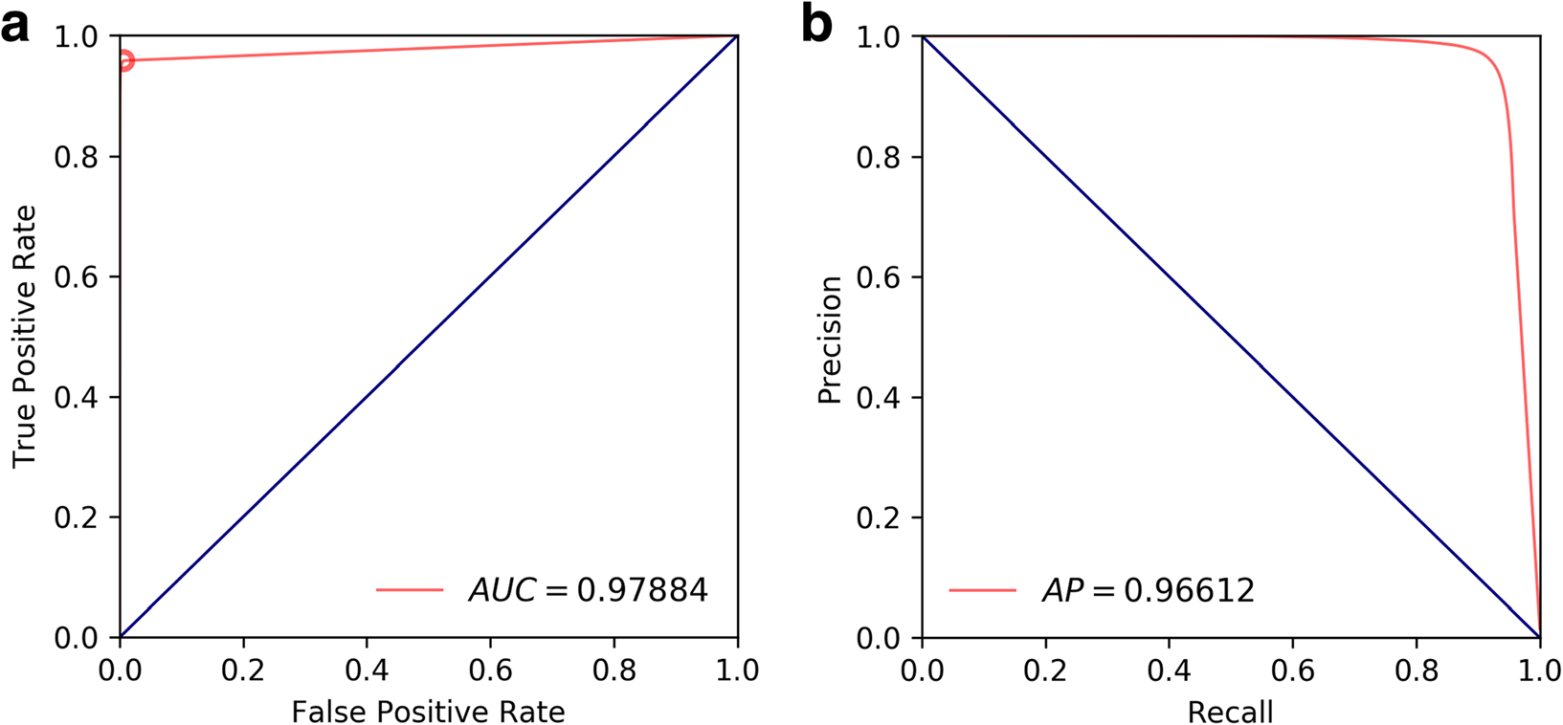
Pixel-level pneumothorax segmentation accuracy evaluation. **a** Receiver operating characteristic curves and corresponding area under curve value (specified in parentheses). **b** Precision-recall curve and corresponding average precision value (specified in parentheses)

Figure 3 shows ROC and precision-recall curves based on pixel-level pneumothorax predictions and corresponding ground-truth annotations. Table 2 provides sensitivity and specificity values evaluated at the Youden Index [21], which is an optimal cut-off point on the ROC curve that simultaneously maximises sensitivity and specificity. Table 2 provides clinical measures, which were calculated based on the precision-recall curve.

**Table 2:** Accuracies for the segmentation of a pneumothorax at the pixel level, detection of a pneumothorax at the volume level, and between radiologists, is displayed in terms of sensitivity, specificity calculated at the Youden index of the receiver operating characteristic curve and corresponding area under curve as well as precision, recall, and Dice similarity coefficient calculated at the optimal cut-off point of the precision-recall curve, and corresponding average precision

Accuracy results			
	Pixel-level segmentation	Inter-rater segmentation	Volume-level detection
Sensitivity	0.958	0.904	0.916
Specificity	0.994	0.999	0.930
Area under the curve	0.979	–	0.976
Precision	0.961	0.944	0.886
Recall	0.919	0.904	0.880
Dice similarity coefficient	0.939	0.924	0.883
Average precision	0.966	–	0.954
Number	9	2	567

**Fig. 4:**
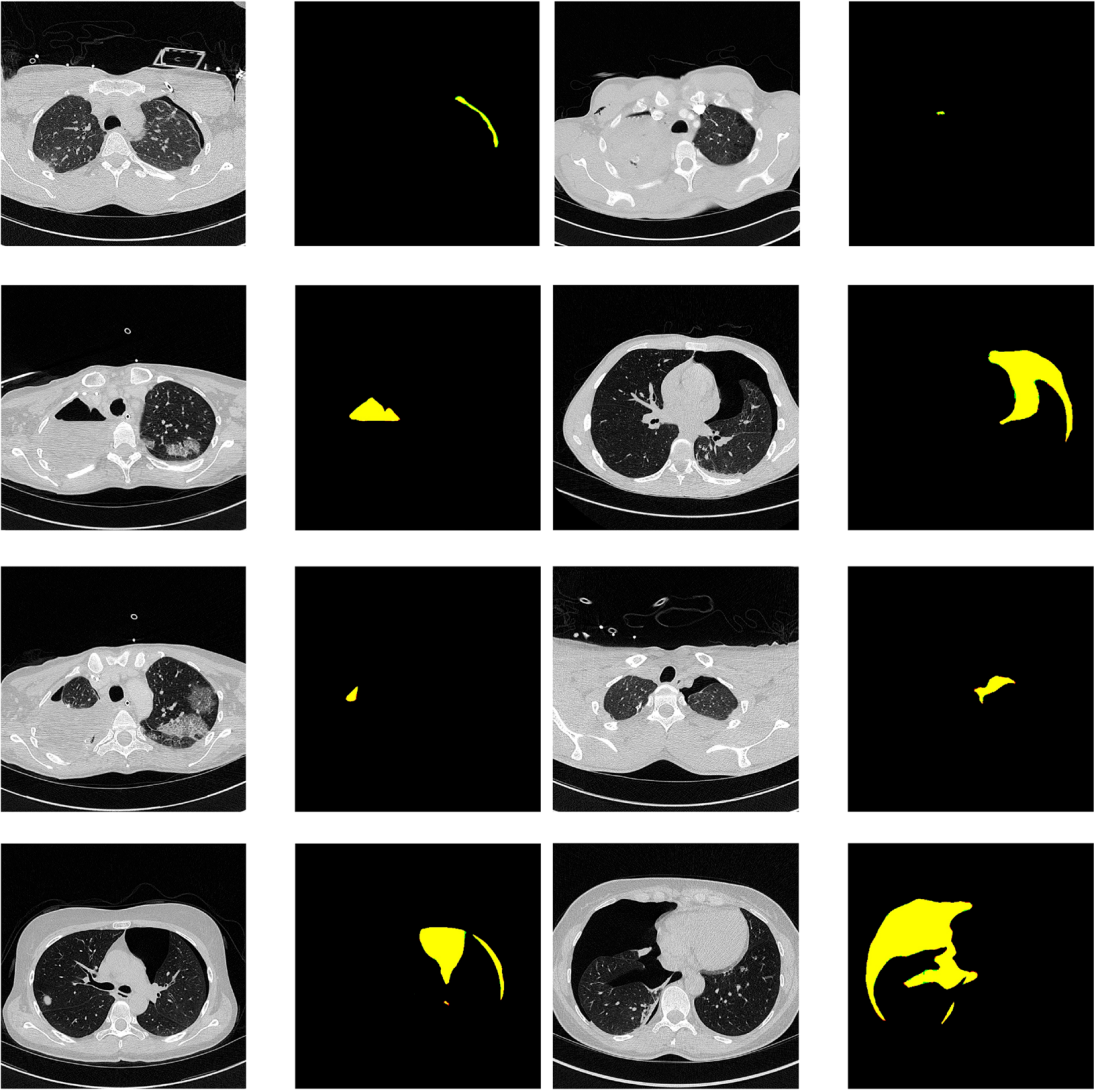
Segmentation results. Raw images and corresponding segmentation results. False-negatives (red), false-positive pneumothorax predictions (green), and correct pneumothorax predictions (yellow) The areas of false prediction are very subtle and correspond to a few pixels on the edges of the correct yellow predictions. In the next Figure (Fig. 5), in contrast, the areas of false prediction are shown more prominently

**Fig. 5:**
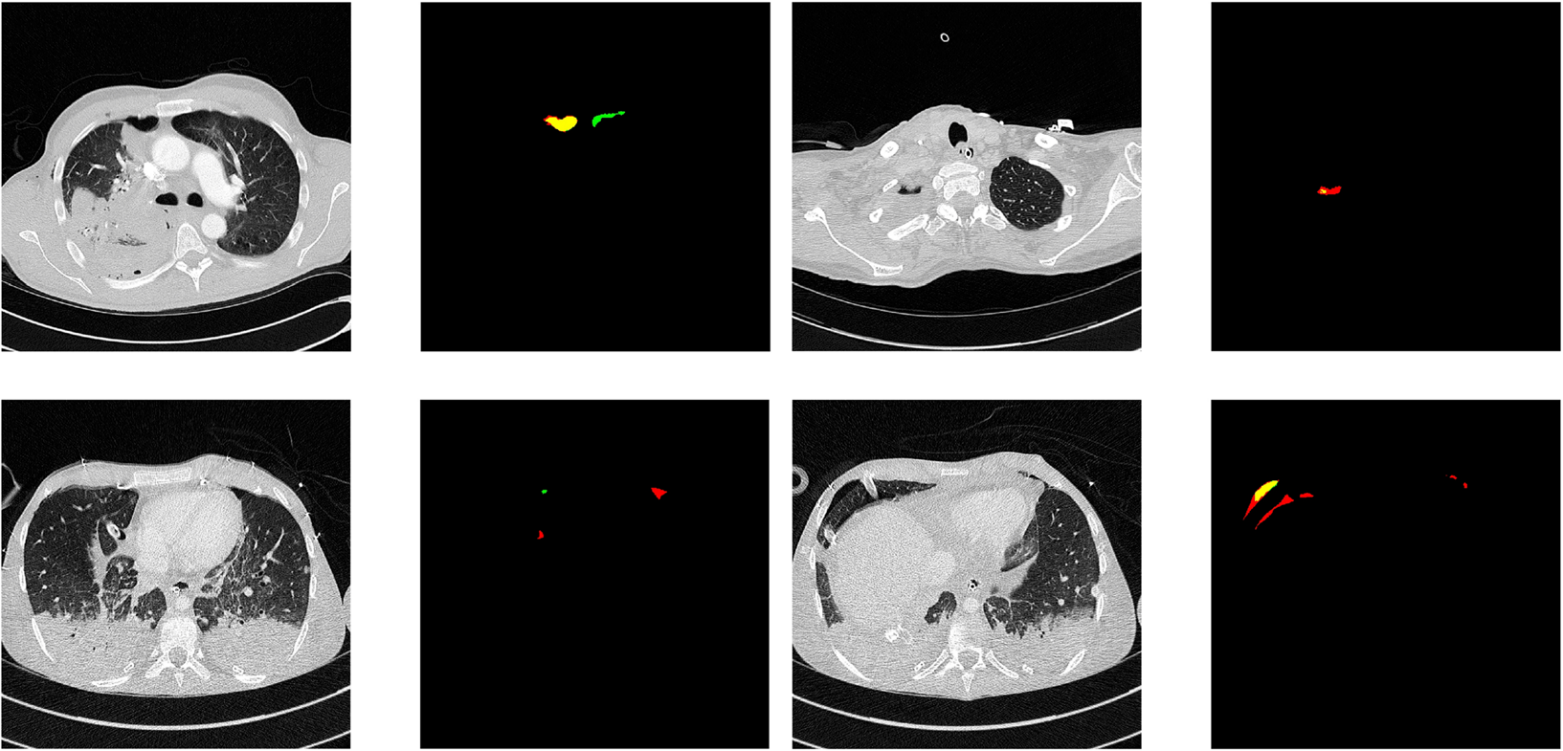
Failure cases of segmentation results. Raw images and corresponding segmentation results. False-negatives (red), false-positive pneumothorax predictions (green), and correct pneumothorax predictions (yellow)

Qualitative segmentation results are shown in Figs. 4 and 5. False-negative cases were observed only in very small pneumothoraxes, in small and thin regions of free air next to chest tubes. False-positive cases were mainly due to panlobular or chest-wall emphysema, bullae, and, in one case, due to misclassification of air inside the main bronchus.

**Fig. 6:**
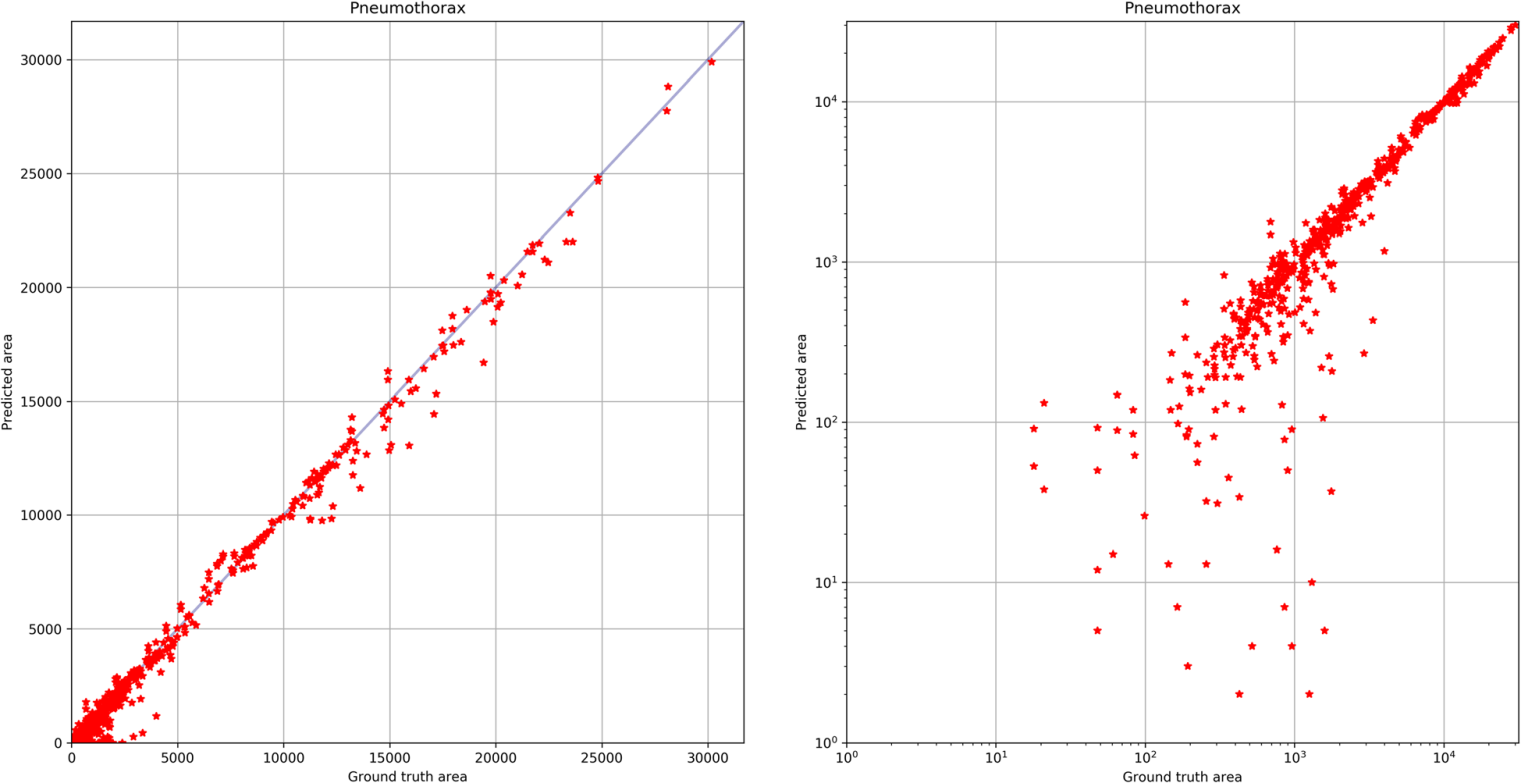
Correlation of predicted and manually assessed pneumothorax areas. Linear scale (left) and log-log scale (right)

Figure 6 shows correlation plots of slice-wise segmented *versus* annotated pneumothorax areas, which evaluated the pneumothorax area estimation accuracy. Calculation of the Pearson correlation coefficient (0.99), *R*^2^ (0.99), and the corresponding two-tailed *p* value (< 0.001) suggested a high linear correlation between predicted and manually delineated pneumothorax areas, with better results for larger pneumothoraxes than for smaller ones. In addition, for each individual pneumothorax occurrence, we evaluated the relative residual, *i.e.,* the absolute difference between predicted and manually delineated pneumothorax area divided by the pneumothorax area. On the test set, the proposed method gave a mean relative residual of 0.14 with a variance of 0.12.

#### Interrater variability evaluation

**Fig. 7:**
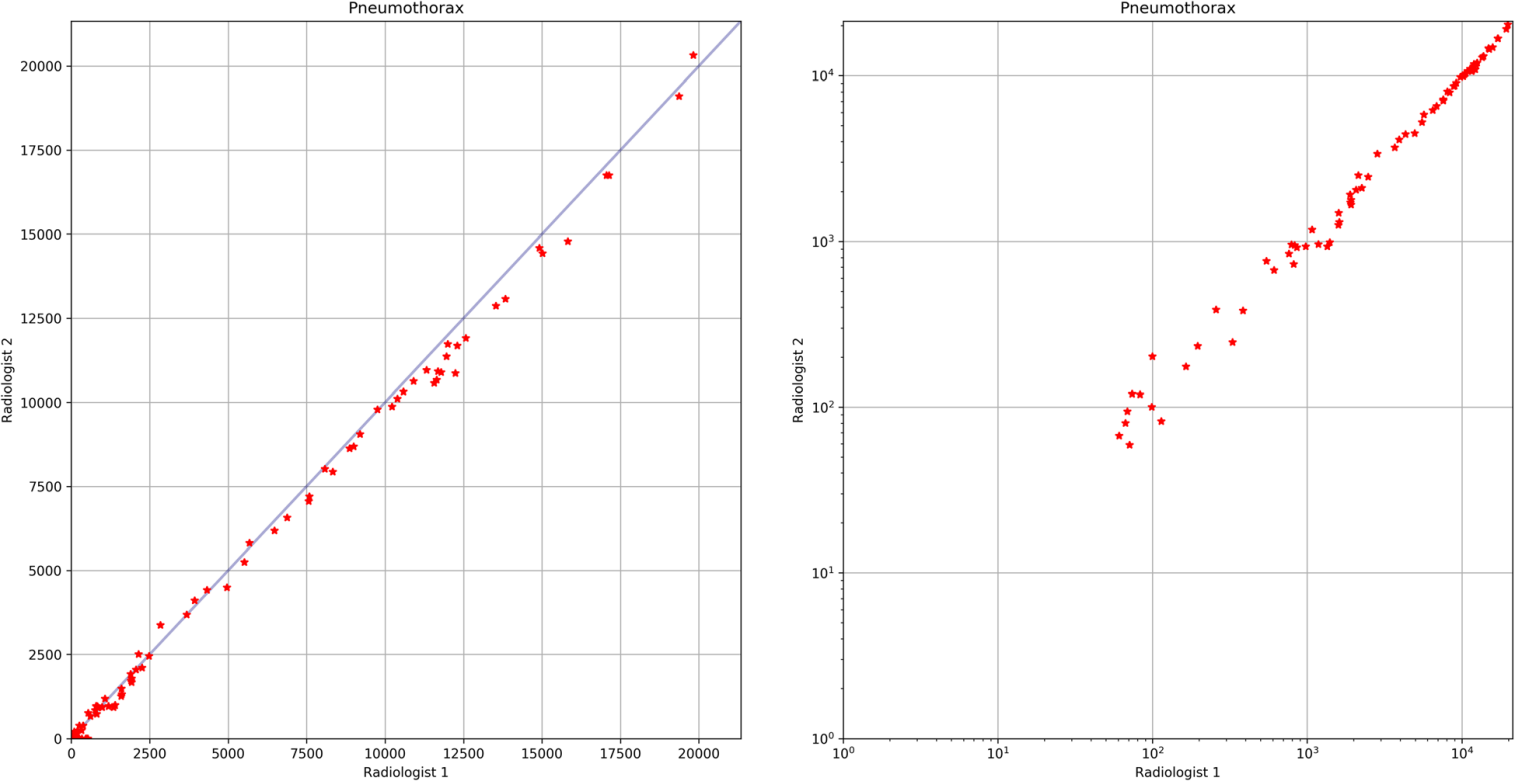
Correlation of slice-wise accumulated pneumothorax areas based on pixel-level pneumothorax annotations independently performed by two radiologists. Linear scale (left) and log-log scale (right)

Table 2 provides precision, sensitivity, specificity, and the DCS, which measured the consensus between two independent annotators in pixel-level pneumothorax identification. Figure 7 shows correlation plots of slice-wise accumulated pneumothorax areas based on pixel-level pneumothorax annotations, independently performed by two radiologists, which evaluate the interrater variability of pneumothorax area quantification. Calculation of the Pearsons correlation coefficient (0.99) and the corresponding two-tailed *p* value (< 0.001) suggested a high linear correlation between predicted and manually delineated pneumothorax areas. These values are comparable to the quantification results obtained by the proposed automated model.

### Volume-level grading accuracy

**Fig. 8:**
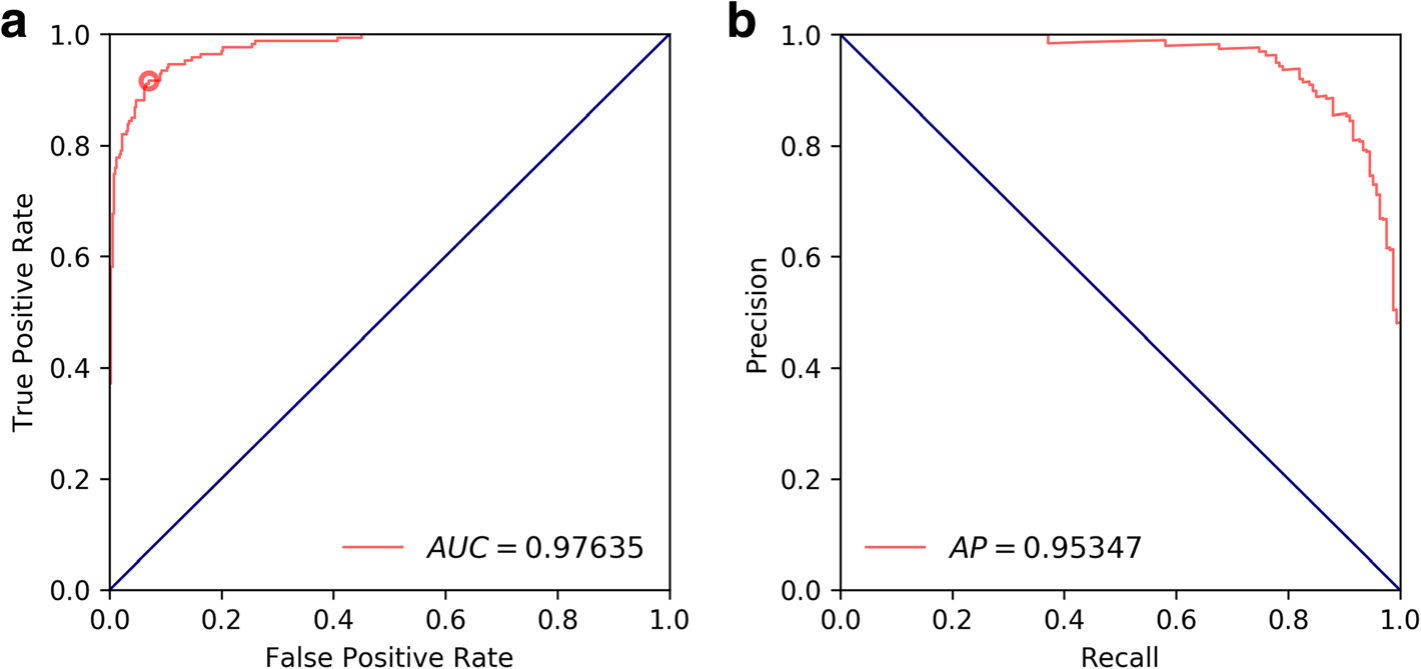
Volume-level pneumothorax detection accuracy evaluation. **a** Receiver operating characteristic curves and corresponding area under curve value (specified in parentheses). **b** Precision-recall curve and corresponding average precision value (specified in parentheses)

Figure 8 shows ROC and precision-recall curves based on volume-level pneumothorax predictions and corresponding ground-truth labels. Table 2 provides sensitivity and specificity values evaluated at the Youden Index. Table 2 provides clinical measures, which were calculated based on the precision-recall curve.

### Execution time

The execution times depend on the number of slices of the CT scans. The computations on volumes of the test set took on average approximately 58 s per volume (minimum 17 s, maximum 80 s).

## Discussion

In this work, we propose and evaluate a method for the accurate classification and quantification of pneumothorax using a deep learning algorithm in a large set of heterogeneous routine chest CT scans that may be utilised in the clinical routine for automated triage of patients. By including scans with pneumothorax that also had additional pathologies from across several CT vendors with a wide variety of technical parameters, we evaluated the algorithm in a more realistic situation than other studies that excluded scans when the pneumothorax was not the only pathology [9]. While previous studies have suggested the use of specific reconstruction parameters to achieve the best results for their computerised pneumothorax volume quantification [11], there is still a large variability regarding the technical setup of chest CT scans in the clinical routine, necessitating the development of flexible and robust solutions.

Our application provides several advantages. Radiologists routinely quantify pneumothorax by measuring the anteroposterior diameter at a representative ventral location, similar to measuring a pneumothorax in chest x-ray radiography [22]. Still, there is a wide variance about what size pneumothorax should be considered small or large. For different modalities, guidelines for the management of pneumothorax report different thresholds, ranging from 15 to 49% of lung volume [23]. Some authors have suggested a size of 15% of the lung volume as a threshold by which to decide between surgical and conservative treatment, as the recurrence of smaller pneumothoraxes treated conservatively was lower than that of those treated with a chest tube, while the time needed for full recovery increased steeply when the pneumothorax was larger than 15 %[24, 25]. Another more recent study suggested 30 mL as a cut-off [5]: whereas smaller ones (< 30 mL) may be treated conservatively, those larger than 30 mL mandate the placement of a chest tube [5].

Measuring pneumothorax size on a chest x-ray is fast, but may be inaccurate compared to the volume estimation on a CT scan [21]. Manual volume assessment on CT scans, however, is very time-consuming and not feasible in clinical practice, necessitating the automation. Moreover, the automatic segmentation and quantification of the thoracic cavity and the pneumothorax would allow the calculation of different measures (*i.e.,* absolute pneumothorax volume *versus* relative volume compared to the size of the thorax or the ipsilateral lung) to determine the measurement with the best predictive performance.

Further, we compared the segmentations of two radiologists in 86 slices to evaluate the reliability of manual segmentations. Taken together, the algorithmic segmentations were able to outperform human segmentations in terms of reliability, indicating that an automatic segmentation would lead to a more consistent, and, thus, more reproducible quantification of free pleural air volume. In this manner, by adding information about the estimated volume of a pneumothorax to the patient’s symptoms and clinical state, the confidence with a therapeutic decision may be improved.

We identified several articles that have reported on problems similar to that of the present study. Cai et al. [10, 11] published specifically tailored models to automatically quantify pneumothorax volume on CT scans of trauma patients and children. They achieved perfect sensitivity by detecting 100% of all cases with a pneumothorax; however, specificity ranged from 10 to 100% in the first study [10]. The second study [11] reported a specificity of 91%. Similar results were reported by a recent study [13], with an overall accuracy of 97%, a sensitivity of 100%, and a specificity of 83%. A high sensitivity is important for detecting all cases of a relevant condition to avoid detrimental consequences. A high specificity, however, is important to avoid radiological follow-up on too many false-positive cases, and, therefore, draw attention from more important findings. Results from the test set suggest that our proposed deep learning method is able to segment a pneumothorax in chest CT scans with high accuracy. In our study, we achieved an AUC of 97.6%, with a sensitivity of 91.6% and a specificity of 93%. False-negative cases can be attributed to very small and thin regions of free air inside the pleural cavity, whereas false-positive cases were mainly due to panlobular or chest-wall emphysema, similar to what the other studies reported.

Another use for the fully automatic detection and quantification of pneumothorax is reflected by the recent advances in the field of ultra-low-dose chest CT scans, which may provide additional information compared to conventional chest x-ray radiography [26]. Due to the limitations of other modalities in estimating pneumothorax volume, an ultra-low-dose chest CT scan would constitute an ideal way to provide follow-up on a pneumothorax. A widespread clinical implementation would lead to a large increase in CT scans, necessitating the triage and automatic quantification to support the radiologists’ workflow, especially in an emergency department situation.

With regard to execution time for one case, our proposed method (on average, 53 s) was comparable to other studies (average of 51 s [9]) or faster (average of 3.1 min [10] and 4 min [11]).

We recognise several limitations of our study. The automated model was trained on interpolated annotations, which led to some smaller variations, such as the fit of the interpolated segmentations resembling the manual annotations, as determined by visual validation. A second limitation is the lower prediction accuracy of smaller pneumothorax volumes (< 27 mL, as estimated by summarising the voxels that were classified as a pneumothorax by the algorithm, see Fig. 6). In these cases, the predicted volume ranged below the manually segmented volume, possibly due to a relatively higher over-segmentation by human readers. However, because the cut-off of 30 mL was used as a guide for the decision between conservative or surgical treatment [5] and both the manual and predicted volume remained below the threshold of 30 mL, there would be no change of therapy based on this discrepancy of volume estimation. A selection bias could be due to the single-centre study design. Our hospital focuses on thoracic surgery and lung transplantation, reflected by the pulmonary primary diagnoses of ‘post-surgical complications’ and ‘lung transplantation’. At other institutions, there might be a different distribution of diagnoses and pathologies, thus leading to a difference in algorithm performance.

In summary, we demonstrated the applicability of a deep learning algorithm for pneumothorax detection and quantification in a large and very heterogeneous cohort of patients from the clinical routine with a wide variety of pathologies. The algorithm-based estimation of pneumothorax size may be used for triage of urgent examinations, to guide clinical decision-making, or to automatically sort and label large amounts of CT scans based on the presence and volume of a pneumothorax for further processing.

## Data Availability

The datasets generated and/or analysed during the current study are not publicly available due to its sensitive nature but are available from the corresponding author on reasonable request.

## Abbreviations

AUC: Area under the curve
CT: Computed tomography
DSC: Dice similarity coefficient
ROC: Receiver operating characteristic
